# Occipital cortex and cerebellum gray matter changes in visual snow syndrome

**DOI:** 10.1212/WNL.0000000000010530

**Published:** 2020-09-29

**Authors:** Francesca Puledda, Muriel Bruchhage, Owen O'Daly, Dominic Ffytche, Steven C.R. Williams, Peter J. Goadsby

**Affiliations:** From the Headache Group, Department of Basic and Clinical Neuroscience (F.P., P.J.G.), Centre for Neuroimaging Sciences, Department of Neuroimaging (M.B., O.O., S.C.R.W.), and Department of Old Age Psychiatry, Institute of Psychiatry, Psychology & Neuroscience (D.F.), King's College London; NIHR–Wellcome Trust King's Clinical Research Facility, SLaM Biomedical Research Centre (F.P., P.J.G.), King's College Hospital, London, UK; and Advanced Baby Imaging Laboratory (M.B.), Warren Alpert School of Medicine at Brown University, Providence, RI.

## Abstract

**Objective:**

To determine whether regional gray and white matter differences characterize the brain of patients with visual snow syndrome, a newly defined neurologic condition, we used a voxel-based morphometry approach.

**Methods:**

In order to investigate whole brain morphology directly, we performed an MRI study on patients with visual snow syndrome (n = 24) and on age- and sex-matched healthy volunteers (n = 24). Voxel-based morphometry was used to determine volumetric differences in patients with visual snow. We further analyzed cerebellar anatomy directly using the high-resolution spatially unbiased atlas template of the cerebellum.

**Results:**

Compared to healthy controls, patients with visual snow syndrome had increased gray matter volume in the left primary and secondary visual cortices, the left visual motion area V5, and the left cerebellar crus I/lobule VI area. These anatomical alterations could not be explained by clinical features of the condition.

**Conclusion:**

Patients with visual snow syndrome have subtle, significant neuroanatomical differences in key visual and lateral cerebellar areas, which may in part explain the pathophysiologic basis of the disorder.

Visual snow syndrome (VSS) is a chronic neurologic condition in which the chief symptom is a constant perception of small moving dots occupying the entire visual field.^1^ Other symptoms include palinopsia, photophobia, entoptic phenomena, and nyctalopia,^2^ which are experienced in different combinations within the syndrome. Visual snow can vary in severity, and it is most disabling when it is accompanied by comorbidities such as migraine and tinnitus.^3^

The pathophysiology of VSS is largely unknown. By investigating brain metabolism in patients with visual snow, an [^18^F]-FDG PET study^4^ revealed significant hypermetabolism in the lingual gyrus as well as a trend of increased metabolism in the left cerebellum.

Gross neuroanatomical abnormalities, as detected by standard clinical neuroimaging, should be excluded for the diagnosis of visual snow, as this is not a “secondary” neurologic condition.^2^ Nonetheless, it is unclear whether subtle morphologic differences might be in part driving, or perhaps be caused by, the disorder.

Using a voxel-based whole-brain morphometry (VBM) approach, we studied the neuroanatomical differences between patients with VSS compared to healthy volunteers. We also directly investigated cerebellar anatomy using the high-resolution atlas template SUIT: a spatially unbiased atlas template of the cerebellum and brainstem.^5,6^ We hypothesized that anatomical differences in VSS would involve the visual network, in particular the primary and secondary visual cortices (areas V1/V2), the precortical visual pathways, as well as the visual motion processing area V5 and the cerebellum. Here, areas showing metabolic alterations with [^18^F]-FDG PET, could potentially present morphologic gray matter (GM) and white matter (WM) differences in patients with VSS.

## Methods

### Population and recruitment

Twenty-four patients with a diagnosis of VSS^2^ and an equal number of age- and sex-matched healthy volunteers were selected for the study. We recruited patients by email, reapproaching patients who had previously contacted our study team asking to participate in research studies. Healthy volunteers were recruited through internal advertisement at King's College London.

Inclusion criteria for participants were age 20–60 years, no contraindications to MRI, no serious medical conditions including psychiatric comorbidities (as assessed by a trained neurologist), no recurrent use of medications with an action on the CNS, and no previous use of any recreational drugs. Healthy volunteers were selected based on matching age (±5 years) and sex of our patient population and had no ongoing medical condition or medication use.

The study involved a telephone interview by a neurologist to assess eligibility of participants, and either 1 or 2 visits to our research facility, depending on whether the participant was in the control or patient group, respectively. A full medical history and general and neurologic examinations were performed for each participant.

### Standard protocol approvals and patient consents

All participants gave their informed consent. The study was approved by the London–City & East Research Ethics Committee (reference number 16/LO/0964).

### MRI

MRI scanning took place on the second visit for patients and the first for volunteers, and lasted approximately 70 minutes. All participants were scanned between 9 and 12 am and told to consume a light breakfast and to avoid caffeine on the morning of the visit. The scanning protocol was the same for both groups and was conducted over a single session. All scans were performed on a 3T General Electric (Cleveland, OH) MR750 MRI scanner at the NIHR–Wellcome Trust King's Clinical Research Facility, King's College Hospital, London, UK, using a 12-channel head coil. High-resolution 3D T1-weighted inversion recovery spoiled gradient echo images were acquired with the following parameters: repetition time 7.312 ms, echo time 3.016 ms, inversion time 400 ms, field of view 270 mm, matrix 256 × 256, slice thickness 1.2 mm, voxel dimension 1.05 × 1.05 × 1.2 mm, 196 slice partitions, array spatial sensitivity encoding technique factor 1.75, in-plane resolution 1 mm.^7^

### VBM with diffeomorphic anatomical registration through exponentiated lie algebra (DARTEL)

Prior to analysis, raw T1 images were visually inspected for artefacts and structural abnormalities that could interfere with the analysis. None of the acquired images was discarded.

MRI data were processed and analyzed using the Statistical Parametric Mapping software suite, version 12 (SPM 12; fil.ion.ucl.ac.uk/spm/) on a MATLAB platform (MATLAB R2017a; uk.mathworks.com/).

VBM analysis to localize regional differences in GM and WM volume was first conducted by applying DARTEL algorithm following the default parameters.^8^ This is a well-accepted automated method for VBM that achieves a more precise registration of individual brain images. In essence, DARTEL allows avoidance of biased image registration by creating an intermediate study-specific template based on the brains of the participants in the study, and by modeling the spatial deformations through a single velocity field that is constant over unit time.

The first step of the procedure segmented each participant's image into GM, WM, and CSF. The second step created a DARTEL population template derived from nonlinear deformation fields for the segmentation procedure and registers all individual deformations to the DARTEL template. In the next registration step, a nonlinear warping of the segmented images allowed to register the DARTEL template in Montreal Neurological Institute space. Furthermore, the voxel values in the tissue maps were modulated by the Jacobian determinant calculated during spatial normalization. Total intracranial volume (TIV) was calculated for each participant within MATLAB from the GM, WM, and CSF tissue components. Finally, all modulated and normalized GM and WM segments were smoothed with full width at half-maximum isotropic Gaussian kernel of 8 mm.

### Structural cerebellar analysis using SUIT

To analyze regional cerebellar volumes, we used the SUIT toolbox version 3.3 (fil.ion.ucl.ac.uk/spm/ext/) within SPM12. This toolbox provides a high-resolution atlas template of the human cerebellum and brainstem that preserves the anatomical detail of cerebellar structures, as well as dedicated procedures to isolate automatically cerebellar structures from the cerebral cortex and to normalize accurately cerebellar structures to this template. Prior to normalization, the individually created isolation maps were loaded into FSLView (fmrib.ox.ac.uk/fsl/), where they were visually inspected against the cropped image and hand corrected if necessary. Using the inverse of the resulting normalization transform, a parcellation of the cerebellum was obtained, based on the probabilistic magnetic resonance atlas of the human cerebellum^6^ provided within the SUIT toolbox. Volumes of interest were then overlaid onto each individual participant's structural scan and inspected to ensure accurate registration.

### VBM analyses

VBM analyses included whole-brain and parcellated cerebellar GM and WM analyses, as well as region of interest (ROI) GM analyses.

For the whole-brain voxel-wise analysis, GM and WM volumes between subgroups of patients with VSS and controls were reviewed with 2-sample *t* tests at an initial cluster-forming voxel threshold of *p* < 0.001. All results were family-wise error (FWE) corrected for multiple comparisons to *p* < 0.05 using the Gaussian random field theory on the basis of cluster extent. TIV, participant age, sex, handedness, and number of disease years were added as covariates in our model. We ran a second model with migraine history as a covariate, since this condition was not present in the control group. An absolute threshold mask of 0.1 was used on both the GM and WM to avoid possible edge effects around the border between the two.

For the cerebellar analysis, morphologic group differences in parcellated cerebellar GM and WM volumes were assessed using the general linear model. TIV of each participant was entered into the design matrix as a nuisance covariate. Further analyses covarying for age, sex, handedness, number of disease years, and migraine presence were also run. The same voxel-wise significance thresholds and masking as the whole-brain analysis were used.

ROI analyses were carried out in the following visual areas: bilateral primary visual cortex (V1/V2), visual motion processing area V5, and the pulvinar. To create ROIs for these anatomical areas, we used the “wfu_pickupatlas Anatomical Library” (nitrc.org/projects/wfu_pickatlas), as implemented in the SPM toolbox, with the exception of the V5 ROI that was created from the “Juelich Histologic Atlas” within FSLeyes. We also defined 2 a priori ROIs based on coordinates from the previous [18F]-FDG PET study on visual snow.^4^ These were the right lingual gyrus (x = 16, y = −78, z = −5) and the left cerebellum (x = −12, y = −62, z = −9); the coordinates were used for small volume correction with a sphere of radius 10 mm. Statistical inferences were made at voxel-wise peak level *p* < 0.05 with FWE correction for multiple comparisons within all the voxels of the ROI. As the cerebellar volumes from SUIT were already parcellated to a small anatomical region, ROI analyses were not performed on these images.

Descriptive statistics and correlation analyses for this study were performed with SPSS Statistics version 24.0 for Windows (IBM, Armonk, NY; spss.com). Pearson correlation coefficients were used to analyze relationships between continuous variables. *p* < 0.05 Was considered significant.

### Data availability

The data that support the findings of this study are available from the study team upon reasonable request.

## Results

### Demographic and clinical data

There were no significant differences with regards to age (mean ± SD for patients with VSS 28 ± 6 and controls 28 ± 5; *p* = 0.8), sex (female:male ratio for patients with VSS 12:12 and controls 14:10; *p* = 0.6), or handedness (right:left ratio for patients with VSS 21:3 and controls 23:1; *p* = 0.3) between the 2 groups. Demographic characteristics and clinical features of the VSS group are summarized in table 1, along with a list of concomitant medications at the time of the study and presence of migraine comorbidity.

**Table 1:**
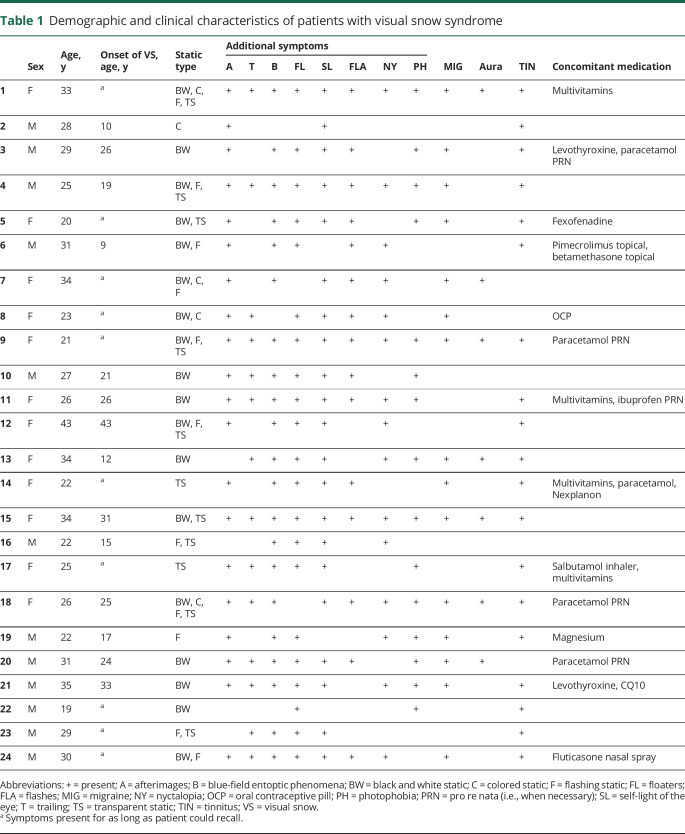
Demographic and clinical characteristics of patients with visual snow syndrome

### Whole-brain VBM-DARTEL analysis

Patients with VSS showed no differences in average total intracranial volume with respect to controls (1,465 ± 113 vs 1,450 ± 146 mL; *p* = 0.6).

**Figure 1:**
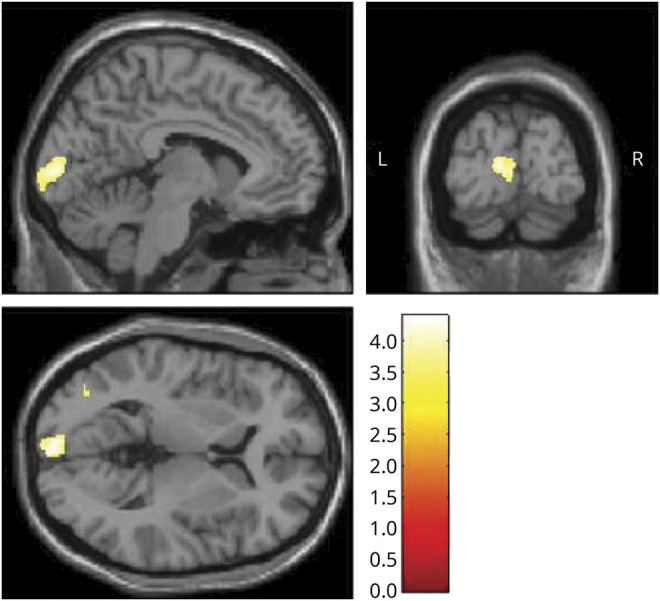
Gray matter (GM) volume increase in the left visual cortex in visual snow syndrome Left primary visual cortex increases in GM volume in patients with VSS with respect to controls (x = −2, y = −98, z = 3; k = 594; *p* = 0.007 uncorrected, *p* = 0.06 family-wise error). Results are from whole-brain analysis; GM volume differences between groups are outlined over T1 images. Bar represents T values.

A whole-brain voxel-wise GM analysis revealed a cluster of increased GM in patients with VSS with respect to controls in the left primary visual cortex (x = −2, y = −98, z = 3; k = 594; *p* = 0.007 uncorrected, *p* = 0.06 FWE corrected; figure 1). When the cluster forming threshold was reduced to *p* = 0.005, this area was significant (*p* = 0.02 FWE corrected). Further, when lowering the threshold to *p* = 0.01 for exploratory purposes, the significant cluster appeared to extend to the homologous region of the contralateral side as well.

No significant differences in WM volumes were found between the 2 groups.

### ROI analysis of GM volumes

Our ROI analyses showed a significant GM volume difference in the left V1/V2 area (main cluster: x = −3, y = −94, z = 0; k = 22; *p* = 0.04 FWE) analogous to the cluster from the whole-brain analysis, as well as in the left V5 area (x = −38, y = −75, z = 4; k = 32; *p* = 0.04 FWE) in VSS. When covarying for migraine presence, both of these areas survived significance.

No significant GM volume differences were found for the remaining ROIs.

### Cerebellar analysis with SUIT

Cerebellar images for one control participant had to be discarded due to poor image quality.

**Figure 2:**
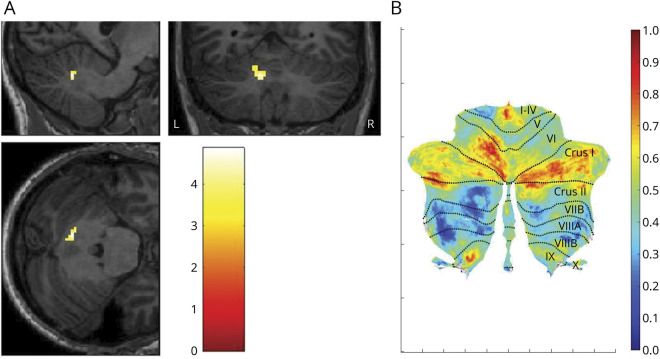
Changes in cerebellar gray matter (GM) volume in visual snow syndrome (VSS) (A) Area of cerebellar gray matter volume increase in patients with VSS (x = −12, y = −62, z = −23; k = 25; *p* = 0.02 family-wise error). GM volume differences between groups are outlined over parcellated cerebellar T1 images. Bar represents *T* values. (B) Cerebellar flatmap of plotted *T* values from patients with VSS with respect to controls (obtained from SUIT within SPM12), with labels for anatomical regions. Bar represents *T* values.

Although the whole-brain VBM analysis did not reveal any specific morphologic differences in the cerebellum of patients with VS, when analyzing the parcellated volumes created with SUIT and corrected by total intracranial volume, we found an area of significant increase of GM volume in crus I/lobule VI of the left cerebellar hemisphere (x = −12, y = −62, z = −23; k = 25; *p* = 0.02 FWE; figure 2). When correcting for age, sex, handedness, migraine, and duration of disease, this area was not significant. However, upon removing one variable at a time from our model, we were able to determine that the area of increased GM was present when we covaried for age and handedness and was significant at a reduced statistical threshold of *p* = 0.005 for the covariates of sex and presence of migraine; finally, it was not significant even at reduced thresholds for the covariate number of disease years. To characterize this further, we examined the beta values for the cluster and ran a multiple regression analysis in SPM on participants with VS only, finding no significant relationship between the increase in GM volume and number of years with the disease.

We found no significant WM volume differences or GM volume decreases in the cerebellum in patients with VSS with respect to healthy controls.

**Figure 3:**
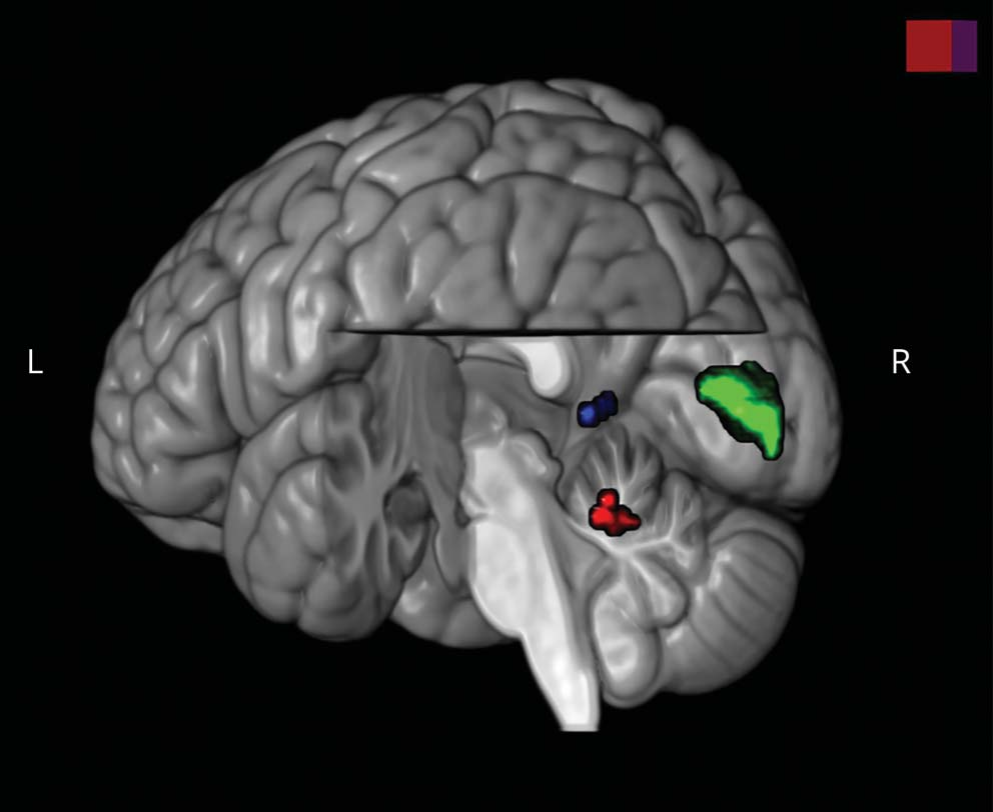
Summary of cortical volumetric changes in patients with visual snow syndrome, as shown by voxel-based morphometry Render illustration of the 3 brain regions of increased gray matter volume in patients with visual snow syndrome with respect to healthy controls. Left V1 cluster is illustrated in green; left V5 cluster in blue; left cerebellum cluster in red. For statistical values of each area, see the text. Image was created in MRICroGL and superimposed on standard brain template.

A summary of all significant areas of GM volume increase in patients with VSS can be found in table 2 and figure 3.

**Table 2:**
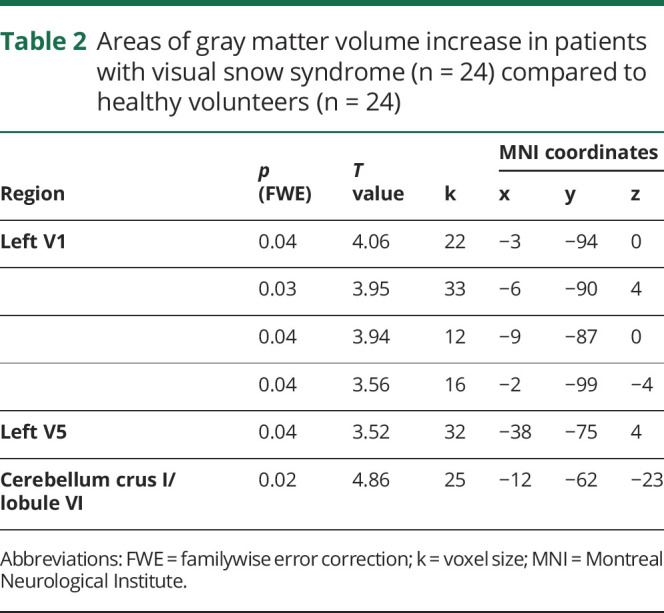
Areas of gray matter volume increase in patients with visual snow syndrome (n = 24) compared to healthy volunteers (n = 24)

### Correlations with clinical features

We extracted the contrast tissue volume estimate values from the left V1, left V5, and cerebellar a priori defined ROIs in all participants and correlated these values with the following variables: age, sex, handedness (in both groups), migraine presence, sum of associated visual symptoms, and disease years (in patients only). No significant correlation was found.

## Discussion

The main finding of our study was that patients with VSS exhibit morphologic changes in GM volume of the left occipital cortex and cerebellum when compared with matched controls. Overall, the results seem to confirm the presence of CNS changes in VSS, underscoring the fundamentally biological basis of the problem. Given that the anatomical changes were not associated with clinical features, such as the total number of disease years and an index of VSS severity, they may represent an inherent trait of visual snow, rather than a consequence of the condition.

The morphologic differences emerging from our analysis involving the primary visual cortex (figure 1) and the visual motion network are in line with the perception of a moving, pan-field visual illusion in VSS. These areas also emerged from our whole-brain analyses, confirming their importance in this neurologic syndrome, and configuring it as a disorder of complex visual processing.

The further involvement of cerebellar areas widely connected with frontal neocortical regions (figure 2) suggests that aside from a sensory dysfunction of visual perception, VSS could also represent a broader network-type disorder, in which more complex alterations of cognitive processing and integration of internal and external stimuli are at play. The analyses including clinical covariates of interest showed that duration of disease in years was the only variable for which this cerebellar area was absent, possibly because of some degree of shared variance between the covariate and the increased GM volume, which might have rendered the result nonsignificant. Nonetheless, it must be noted that this effect was not explained by the duration of disease itself. Further, the reliability of number of years with the condition could be susceptible to recall biases, possibly hindering its validity as a clinical measure.

In patients with VSS, there were no significant structural differences in the lingual gyrus, where metabolic alterations were found previously with [18F]-FDG PET.^4^

Considering that VSS is a disorder linked to alterations in brain function, the absence of major changes in morphology was expected. Conversely, an altered structure of primary and secondary visual areas is in keeping with the clinical experience of simple visual illusions typical of visual snow.^9^ Furthermore, GM increases in the primary visual cortex very clearly followed the calcarine fissure (figure 1), showing a correspondence to the retinotopic mapping of the entire hemifield.

The fact that the morphologic V1 change was only found in the left side is more difficult to interpret. It is possible that this finding was due to a statistical issue, rather than to a truly lateralized morphologic difference, given that a GM volume increase was also present in the same region on the right side when lowering the significance threshold. It is unlikely this was due to handedness, as this variable was corrected for in the analysis. Interestingly, we found no morphologic alterations of precortical visual pathways in this study.

The visual motion network spreads from V1 dorsally to the parietal lobe, encompassing visual motion area V5, which is located in the ventrolateral temporo-parietal-occipital junction and specifically responds to motion stimuli.^10^ This area is involved primarily in decoding information and patterns of direction, speed, and motion.^11^ The motion network is also composed of subcompartments within V1/V2, of area V3/V3A in the cuneus, and finally of Brodmann area 7 in the precuneus.^12^ It is part of the dorsal stream, now renamed the “how-pathway,”^13^ which integrates information on spatial localization of incoming visual information for the purpose of skilled motor planning. Importantly, area V5 was also identified in a recent study of regional brain perfusion in visual snow.^14^

In addition to differences in neocortical visual networks, we also demonstrated differences in the lateral cerebellum in patients with VSS when compared with controls. The left cerebellar lobule VI showed increased metabolism in a previous [18F]-FDG PET study,^4^ in an area contiguous with the one found in the present study. This constitutes a possible link between the previously documented functional alteration and an underlying structural abnormality in VSS. This region is directly involved in spatial processing functions and in the “preparation” of somatosensory integration.^15^ Most importantly, the cerebellar lobule VI/crus I form part of the so-called “cognitive cerebellum,”^16^ which, owing to widespread cerebello-cortical connections,^17,18^ has a role in complex functions such as language, executive action, and visual working memory.^19^ In particular, these cerebellar subregions have been associated with the frontoparietal and default mode networks (DMNs) in resting-state analyses.^20^ These networks are control systems that work in synergy when the brain is involved in a task or at rest.^21,22^ The frontoparietal network in particular is involved in top-down attentional control and adaptation behaviors,^23,24^ whereas the DMN is responsible for monitoring the internal mental landscape.^25,26^

Several pathophysiologic hypotheses for the genesis of VSS have been proposed in the literature,^27^ and hyperexcitation of primary and secondary visual cortices^28–31^ seems to be one of the most plausible. In a condition where internal visual information is constantly being perceived, a state of increased cortical activation, justified by a form of processing overload, is plausible. We hypothesize that this functional hyperactivation could in turn be causing localized increases in GM volume, such as the ones we found in our study. Interestingly, important pathways involved in integration and processing of visual stimuli as well as action and attention networks were simultaneously affected in VSS, an aspect that will need to be explored in future studies.

A similar disorder of brain function that has been linked to visual snow, both on a clinical and pathophysiologic basis, is migraine.^4^ The high comorbidity between these conditions was confirmed in our population, where more than half the participants presented a history of migraine.

Several studies have shown morphologic GM and WM changes in the visual areas and cerebellum of migraineurs with and without aura. One VBM study in particular showed a GM volume decrease in V5 in migraineurs with respect to controls, which correlated with disease activity^32^; another found decreased GM volume in the left V1/V2 area and cerebellum.^33^ These findings, together with the subanalyses we conducted in which migraine presence was included as a covariate, seem to suggest that our opposite results of increased GM volume in patients with VSS are due to the visual snow condition alone.

The absence of a correlation between morphologic changes and clinical features of VSS is an issue. This may be due to the absence of an objective and reliable instrument to measure the severity of the condition. However, as VSS is a relatively homogenous condition,^3^ and having selected a patient population with similar clinical features, we suggest that the morphologic changes that emerged from this study can be generalized to a larger number of patients. Given that eye dominance was not routinely tested as part of the study, it is not possible to exclude its effect on the laterality of the morphologic changes found within the visual cortex. However, given that GM increases appeared bilaterally when the significance threshold was lowered, it is more likely that this finding was due to low statistical power.

Our study confirms that the neurologic syndrome of visual snow is characterized by subtle anatomic brain changes in affected patients. These morphologic GM alterations involve relevant neocortical visual areas pertaining to motion pathways, as well as important cognitive and attentional cerebellar areas, and seem to represent an inherent trait of the condition. These abnormalities could partially explain the functional changes found through other neuroimaging techniques in VSS, and potentially some of its clinical elements as well. Our results provide further insight into a relatively unknown condition, creating the basis for future investigations in the disorder. This is a necessary step in order to provide optimal treatment strategies for patients, either pharmacologic or neuromodulatory, which are currently lacking.

## Glossary

DARTEL: diffeomorphic anatomical registration through exponentiated lie algebra
DMN: default mode network
FWE: family-wise error
GM: gray matter
ROI: region of interest
SPM: statistical parametric mapping
TIV: total intracranial volume
VBM: voxel-based morphometry
VSS: visual snow syndrome
WM: white matter
